# Supplementary material for: Enhanced skeletal muscle ribosome biogenesis, yet attenuated mTORC1 and ribosome biogenesis-related signalling, following short-term concurrent versus single-mode resistance training
Source: Sci Rep. 2018 Jan 12;8:560. doi: 10.1038/s41598-017-18887-6 (PMC5766515; doi:10.1038/s41598-017-18887-6)
Supplement: Supplementary file 1 — Supplementary Information [file 41598_2017_18887_MOESM1_ESM.pdf]

SUPPLEMENTARY FILE A: Summary of magnitude-based inference (MBI) data for all within-group and between-group comparisons.

Manuscript title: Enhanced skeletal muscle ribosome biogenesis, yet attenuated mTORC1 and ribosome biogenesis-related signalling, following short-term concurrent versus single-mode resistance training.

Author list: Jackson J. Fyfe[1,2,3*], David J. Bishop[1,4], Jonathan D. Bartlett[1], Erik D. Hanson[1,5], Mitchell J. Anderson[1], Andrew P. Garnham[1,2] & Nigel K. Stepto[1,6,7].

Author affiliations:

1) Institute of Sport, Exercise and Active Living (ISEAL), Victoria University, Melbourne, Australia; 2) School of Exercise and Nutrition Sciences, Deakin University, Melbourne, Australia; 3) Centre for Sport Research (CSR), Deakin University, Melbourne, Australia; 4) School of Medical and Health Sciences, Edith Cowan University, Joondalup, Australia; 5) Department of Exercise and Sport Science, University of North Carolina at Chapel Hill, North Carolina, USA; 6) Monash Centre for Health Research and Implementation, School of Public Health and Preventive Medicine, Monash University, Melbourne, Australia; 7) Australian Institute for Musculoskeletal Science (AIMSS), University of Melbourne, Victoria University and Western Health, Sunshine Hospital, St Albans, Australia

Supplementary data Table 1. Summary of magnitude-based inference (MBI) data for all within-group comparisons.

Measure	Group	Comparison	Mean difference		Standardised effect size (ES)		Effect magnitude	Qualitative likelihood of true effect magnitude being substantial	*P* value
			% difference	±90% CL	ES (*d*)	±90% CL			
**p-mTOR^{Ser2448}**	RT	PRE-POST	1	65	0.01	0.39	trivial	very unlikely	0.967
		PRE-+1 h	108	125	0.42	0.33	small	possibly	0.036
		PRE-+3 h	143	130	0.49	0.28	small	possibly	0.022
		POST-+1 h	105	137	0.46	0.40	small	possibly	0.048
		POST-+3 h	140	245	0.56	0.57	small	possibly	0.083
	HIT+RT	PRE-POST	9	31	0.10	0.33	trivial	unlikely	0.557
		PRE-+1 h	42	51	0.38	0.38	small	possibly	0.077
		PRE-+3 h	85	51	0.60	0.27	moderate	likely	0.031
		POST-+1 h	30	71	0.32	0.62	small	possibly	0.320
		POST-+3 h	70	45	0.64	0.31	moderate	likely	0.030
	MICT+RT	PRE-POST	-25	39	-0.18	0.33	trivial	unlikely	0.441
		PRE-+1 h	33	157	0.17	0.59	trivial	unlikely	0.553
		PRE-+3 h	16	67	0.07	0.28	trivial	very unlikely	0.707
		POST-+1 h	77	184	0.37	0.59	small	possibly	0.218
		POST-+3 h	53	46	0.28	0.19	small	unlikely	0.032
**Total mTOR**	RT	PRE-POST	5	9	0.07	0.12	trivial	most unlikely	0.740
		PRE-+1 h	20	28	0.23	0.29	small	unlikely	0.212
		PRE-+3 h	31	52	0.27	0.39	small	possibly	0.245
		POST-+1 h	14	24	0.18	0.28	trivial	unlikely	0.285
		POST-+3 h	25	42	0.30	0.44	small	possibly	0.212

**Measure**	**Group**	**Comparison**	**Mean difference**		**Standardised effect size (ES)**		**Effect magnitude**	**Qualitative likelihood of true effect magnitude being substantial**	***P* value**
			**% difference**	**±90% CL**	**ES (*d*)**	**±90% CL**			
	HIT+RT	PRE-POST	6	15	0.09	0.20	trivial	very unlikely	0.455
		PRE-+1 h	-7	20	-0.08	0.27	trivial	very unlikely	0.533
		PRE-+3 h	-9	32	-0.10	0.36	trivial	unlikely	0.600
		POST-+1 h	-12	16	-0.18	0.26	trivial	unlikely	0.194
		POST-+3 h	-14	24	-0.21	0.39	small	unlikely	0.289
	MICT+RT	PRE-POST	29	37	0.43	0.48	small	possibly	0.104
		PRE-+1 h	17	32	0.23	0.42	small	unlikely	0.375
		PRE-+3 h	11	19	0.18	0.28	trivial	unlikely	0.291
		POST-+1 h	-9	16	-0.17	0.30	trivial	unlikely	0.551
		POST-+3 h	-14	30	-0.25	0.58	small	possibly	0.428
**p-p70S6K1^{Thr389}**	RT	PRE-POST	46	84	0.33	0.48	small	possibly	0.248
		PRE-+1 h	160	58	0.74	0.17	moderate	most likely	< 0.001
		PRE-+3 h	210	191	0.84	0.43	moderate	very likely	0.013
		POST-+1 h	78	77	0.51	0.37	small	possibly	0.026
		POST-+3 h	112	209	0.66	0.76	moderate	possibly	0.115
	HIT+RT	PRE-POST	94	47	0.66	0.24	moderate	very likely	0.024
		PRE-+1 h	113	101	0.71	0.43	moderate	likely	0.065
		PRE-+3 h	117	92	0.62	0.33	moderate	likely	0.029
		POST-+1 h	10	53	0.09	0.47	trivial	unlikely	0.703
		POST-+3 h	12	45	0.11	0.39	trivial	unlikely	0.574

Measure	Group	Comparison	Mean difference		Standardised effect size (ES)		Effect magnitude	Qualitative likelihood of true effect magnitude being substantial	*P* value
			% difference	±90% CL	ES (*d*)	±90% CL			
	MICT+RT	PRE-POST	27	50	0.38	0.60	small	possibly	0.250
		PRE-+1 h	66	26	0.75	0.23	moderate	very likely	0.058
		PRE-+3 h	35	31	0.51	0.39	small	possibly	0.097
		POST-+1 h	31	63	0.42	0.74	small	possibly	0.321
		POST-+3 h	6	38	0.09	0.56	trivial	unlikely	0.734
**Total-p70S6K1**	RT	PRE-POST	3	12	0.06	0.23	trivial	very unlikely	0.602
		PRE-+1 h	-10	12	-0.19	0.24	trivial	unlikely	0.211
		PRE-+3 h	-17	22	-0.30	0.41	small	possibly	0.261
		POST-+1 h	-13	16	-0.27	0.36	small	possibly	0.181
		POST-+3 h	-20	20	-0.44	0.49	small	possibly	0.168
	HIT+RT	PRE-POST	10	13	0.27	0.35	small	possibly	0.277
		PRE-+1 h	-7	15	-0.18	0.40	trivial	unlikely	0.505
		PRE-+3 h	-9	9	-0.22	0.24	small	unlikely	0.091
		POST-+1 h	-15	10	-0.47	0.33	small	possibly	0.020
		POST-+3 h	-17	7	-0.55	0.24	small	likely	0.026
	MICT+RT	PRE-POST	-19	43	-0.22	0.53	small	possibly	0.400
		PRE-+1 h	-9	28	-0.09	0.29	trivial	very unlikely	0.560
		PRE-+3 h	6	17	0.06	0.18	trivial	very unlikely	0.513
		POST-+1 h	13	33	0.13	0.30	trivial	unlikely	0.491
		POST-+3 h	31	74	0.28	0.56	small	possibly	0.332

Measure	Group	Comparison	Mean difference		Standardised effect size (ES)		Effect magnitude	Qualitative likelihood of true effect magnitude being substantial	*P* value
			% difference	±90% CL	ES (*d*)	±90% CL			
**p-rps6$^{Ser235/236}$**	RT	PRE-POST	9	105	0.03	0.31	trivial	very unlikely	0.846
		PRE-+1 h	769	885	0.68	0.28	moderate	very likely	< 0.001
		PRE-+3 h	1059	1361	0.71	0.29	moderate	very likely	< 0.001
		POST-+1 h	700	678	0.75	0.28	moderate	very likely	< 0.001
		POST-+3 h	967	1047	0.85	0.31	moderate	very likely	< 0.001
	HIT+RT	PRE-POST	16	48	0.06	0.15	trivial	most unlikely	0.477
		PRE-+1 h	568	827	0.63	0.35	moderate	likely	0.007
		PRE-+3 h	357	420	0.44	0.24	small	possibly	0.007
		POST-+1 h	475	572	0.66	0.33	moderate	likely	0.005
		POST-+3 h	294	319	0.51	0.28	small	likely	0.006
	MICT+RT	PRE-POST	7	23	0.05	0.16	trivial	most unlikely	0.711
		PRE-+1 h	673	502	1.44	0.43	large	most likely	0.001
		PRE-+3 h	195	158	0.67	0.31	moderate	likely	0.032
		POST-+1 h	621	420	1.49	0.42	large	most likely	< 0.001
		POST-+3 h	176	200	0.76	0.51	moderate	likely	0.026
**Total-rps6**	RT	PRE-POST	-2	12	-0.03	0.22	trivial	very unlikely	0.858
		PRE-+1 h	15	21	0.23	0.31	small	unlikely	0.324
		PRE-+3 h	17	29	0.22	0.35	small	unlikely	0.235
		POST-+1 h	17	21	0.28	0.32	small	unlikely	0.136
		POST-+3 h	19	24	0.31	0.36	small	possibly	0.119

Measure	Group	Comparison	Mean difference		Standardised effect size (ES)		Effect magnitude	Qualitative likelihood of true effect magnitude being substantial	*P* value
			% difference	±90% CL	ES (*d*)	±90% CL			
	HIT+RT	PRE-POST	11	16	0.05	0.07	trivial	most unlikely	0.231
		PRE-+1 h	12	17	0.05	0.07	trivial	most unlikely	0.223
		PRE-+3 h	23	31	0.09	0.11	trivial	most unlikely	0.146
		POST-+1 h	1	9	0.00	0.05	trivial	most unlikely	0.881
		POST-+3 h	11	15	0.05	0.07	trivial	most unlikely	0.163
	MICT+RT	PRE-POST	17	26	0.07	0.10	trivial	most unlikely	0.204
		PRE-+1 h	35	28	0.12	0.09	trivial	most unlikely	0.022
		PRE-+3 h	31	26	0.10	0.08	trivial	most unlikely	0.080
		POST-+1 h	15	27	0.07	0.11	trivial	most unlikely	0.236
		POST-+3 h	12	27	0.05	0.11	trivial	most unlikely	0.364
**p-4E-BP1$^{Thr37/46}$**	RT	PRE-POST	35	32	0.37	0.28	small	possibly	0.216
		PRE-+1 h	26	55	0.28	0.50	small	possibly	0.450
		PRE-+3 h	22	30	0.21	0.26	small	unlikely	0.505
		POST-+1 h	-7	21	-0.09	0.28	trivial	very unlikely	0.535
		POST-+3 h	-10	20	-0.13	0.26	trivial	very unlikely	0.400
	HIT+RT	PRE-POST	-7	20	-0.12	0.36	trivial	unlikely	0.651
		PRE-+1 h	-6	21	-0.10	0.36	trivial	unlikely	0.692
		PRE-+3 h	11	25	0.15	0.34	trivial	unlikely	0.536
		POST-+1 h	1	27	0.01	0.45	trivial	unlikely	0.956
		POST-+3 h	18	37	0.29	0.53	small	possibly	0.289

Measure	Group	Comparison	Mean difference		Standardised effect size (ES)		Effect magnitude	Qualitative likelihood of true effect magnitude being substantial	*P* value
			% difference	±90% CL	ES (*d*)	±90% CL			
	MICT+RT	PRE-POST	21	35	0.29	0.42	small	possibly	0.341
		PRE-+1 h	32	36	0.37	0.36	small	possibly	0.187
		PRE-+3 h	59	53	0.56	0.40	small	likely	0.027
		POST-+1 h	9	26	0.13	0.35	trivial	unlikely	0.461
		POST-+3 h	59	53	0.70	0.49	moderate	likely	0.233
**Total-4E-BP1**	RT	PRE-POST	-3	20	-0.02	0.12	trivial	most unlikely	0.758
		PRE-+1 h	-20	56	-0.11	0.34	trivial	unlikely	0.508
		PRE-+3 h	-27	73	-0.13	0.38	trivial	unlikely	0.488
		POST-+1 h	-17	44	-0.11	0.30	trivial	unlikely	0.474
		POST-+3 h	-24	59	-0.16	0.43	trivial	unlikely	0.449
	HIT+RT	PRE-POST	13	15	0.27	0.28	small	unlikely	0.213
		PRE-+1 h	12	15	0.25	0.28	small	unlikely	0.297
		PRE-+3 h	11	13	0.23	0.25	small	unlikely	0.394
		POST-+1 h	-1	6	-0.02	0.14	trivial	most unlikely	0.770
		POST-+3 h	-2	8	-0.04	0.17	trivial	most unlikely	0.759
	MICT+RT	PRE-POST	8	9	0.11	0.11	trivial	most unlikely	0.137
		PRE-+1 h	10	32	0.12	0.36	trivial	unlikely	0.548
		PRE-+3 h	-3	6	-0.04	0.07	trivial	most unlikely	0.502
		POST-+1 h	2	26	0.02	0.35	trivial	very unlikely	0.896
		POST-+3 h	-11	10	-0.16	0.16	trivial	very unlikely	0.078

Measure	Group	Comparison	Mean difference		Standardised effect size (ES)		Effect magnitude	Qualitative likelihood of true effect magnitude being substantial	*P* value
			% difference	±90% CL	ES (*d*)	±90% CL			
**p-ACC^{Ser79}**	RT	PRE-POST	47	76	0.24	0.30	small	unlikely	0.153
		PRE-+1 h	-6	68	-0.03	0.37	trivial	unlikely	0.854
		PRE-+3 h	-19	40	-0.11	0.24	trivial	very unlikely	0.422
		POST-+1 h	-36	22	-0.28	0.20	small	unlikely	0.026
		POST-+3 h	-45	20	-0.37	0.22	small	possibly	0.012
	HIT+RT	PRE-POST	14	21	0.10	0.14	trivial	most unlikely	0.373
		PRE-+1 h	44	68	0.26	0.33	small	unlikely	0.321
		PRE-+3 h	69	85	0.35	0.32	small	possibly	0.143
		POST-+1 h	27	41	0.18	0.24	trivial	unlikely	0.319
		POST-+3 h	48	82	0.30	0.40	small	possibly	0.196
	MICT+RT	PRE-POST	15	32	0.12	0.25	trivial	very unlikely	0.380
		PRE-+1 h	-38	24	-0.42	0.33	small	possibly	0.089
		PRE-+3 h	-12	22	-0.12	0.23	trivial	very unlikely	0.708
		POST-+1 h	-46	20	-0.56	0.33	small	likely	0.016
		POST-+3 h	-23	26	-0.24	0.29	small	unlikely	0.371
**p-AMPK^{Thr172}**	RT	PRE-POST	29	106	0.15	0.44	trivial	unlikely	0.586
		PRE-+1 h	129	141	0.44	0.31	small	possibly	0.035
		PRE-+3 h	98	189	0.34	0.42	small	possibly	0.082
		POST-+1 h	78	72	0.34	0.23	small	possibly	0.031
		POST-+3 h	166	249	0.58	0.49	small	possibly	0.184

Measure	Group	Comparison	Mean difference		Standardised effect size (ES)		Effect magnitude	Qualitative likelihood of true effect magnitude being substantial	*P* value
			% difference	±90% CL	ES (*d*)	±90% CL			
	HIT+RT	PRE-POST	5	61	0.04	0.41	trivial	unlikely	0.860
		PRE-+1 h	19	69	0.12	0.38	trivial	unlikely	0.578
		PRE-+3 h	-2	42	-0.02	0.28	trivial	very unlikely	0.935
		POST-+1 h	13	93	0.09	0.56	trivial	unlikely	0.747
		POST-+3 h	-7	28	-0.06	0.22	trivial	very unlikely	0.647
	MICT+RT	PRE-POST	42	48	0.32	0.31	small	possibly	0.123
		PRE-+1 h	81	54	0.54	0.27	small	likely	0.046
		PRE-+3 h	17	39	0.15	0.30	trivial	unlikely	0.592
		POST-+1 h	28	47	0.23	0.33	small	unlikely	0.220
		POST-+3 h	-17	12	-0.18	0.13	trivial	very unlikely	0.187
**Total AMPK**	RT	PRE-POST	8	7	0.26	0.20	small	unlikely	0.073
		PRE-+1 h	5	9	0.16	0.24	trivial	unlikely	0.344
		PRE-+3 h	1	13	0.04	0.35	trivial	very unlikely	0.866
		POST-+1 h	-3	9	-0.09	0.30	trivial	very unlikely	0.573
		POST-+3 h	-6	10	-0.21	0.35	small	unlikely	0.296
	HIT+RT	PRE-POST	32	16	0.63	0.28	moderate	likely	0.091
		PRE-+1 h	43	19	0.80	0.29	moderate	very likely	0.029
		PRE-+3 h	12	23	0.22	0.40	small	unlikely	0.536
		POST-+1 h	9	16	0.19	0.33	trivial	unlikely	0.281
		POST-+3 h	-15	11	-0.37	0.30	small	possibly	0.039

Measure	Group	Comparison	Mean difference		Standardised effect size (ES)		Effect magnitude	Qualitative likelihood of true effect magnitude being substantial	*P* value
			% difference	±90% CL	ES (*d*)	±90% CL			
	MICT+RT	PRE-POST	13	19	0.26	0.37	small	unlikely	0.225
		PRE-+1 h	1	12	0.03	0.23	trivial	very unlikely	0.819
		PRE-+3 h	7	19	0.11	0.30	trivial	unlikely	0.448
		POST-+1 h	-10	14	-0.23	0.33	small	unlikely	0.197
		POST-+3 h	-6	9	-0.12	0.19	trivial	very unlikely	0.369
**p-TIF-1A^{Ser649}**	RT	PRE-POST	34	95	0.16	0.37	trivial	unlikely	0.421
		PRE-+1 h	199	151	0.54	0.24	small	likely	0.005
		PRE-+3 h	357	485	0.67	0.41	moderate	likely	0.012
		POST-+1 h	123	79	0.45	0.19	small	possibly	0.002
		POST-+3 h	241	315	0.69	0.46	moderate	likely	0.017
	HIT+RT	PRE-POST	133	102	0.62	0.31	moderate	likely	0.047
		PRE-+1 h	211	75	0.76	0.16	moderate	most likely	0.034
		PRE-+3 h	283	268	0.79	0.38	moderate	very likely	0.006
		POST-+1 h	33	65	0.21	0.34	small	unlikely	0.301
		POST-+3 h	64	80	0.36	0.34	small	possibly	0.108
	MICT+RT	PRE-POST	35	54	0.26	0.34	small	unlikely	0.255
		PRE-+1 h	77	83	0.45	0.36	small	possibly	0.075
		PRE-+3 h	16	68	0.11	0.40	trivial	unlikely	0.645
		POST-+1 h	31	103	0.23	0.62	small	possibly	0.434
		POST-+3 h	-13	48	-0.12	0.46	trivial	unlikely	0.571

Measure	Group	Comparison	Mean difference		Standardised effect size (ES)		Effect magnitude	Qualitative likelihood of true effect magnitude being substantial	*P* value
			% difference	±90% CL	ES (*d*)	±90% CL			
**Total-TIF-1A**	RT	PRE-POST	-1	17	-0.02	0.22	trivial	very unlikely	0.866
		PRE-+1 h	-28	25	-0.38	0.39	small	possibly	0.108
		PRE-+3 h	-43	19	-0.56	0.32	small	likely	0.010
		POST-+1 h	-27	18	-0.41	0.32	small	possibly	0.042
		POST-+3 h	-42	19	-0.70	0.42	moderate	likely	0.010
	HIT+RT	PRE-POST	-16	12	-0.17	0.14	trivial	very unlikely	0.047
		PRE-+1 h	-24	17	-0.24	0.20	small	unlikely	0.059
		PRE-+3 h	-42	17	-0.41	0.21	small	possibly	0.038
		POST-+1 h	-9	25	-0.10	0.27	trivial	very unlikely	0.471
		POST-+3 h	-32	28	-0.38	0.40	small	possibly	0.203
	MICT+RT	PRE-POST	-5	23	-0.03	0.14	trivial	most unlikely	0.684
		PRE-+1 h	-59	74	-0.44	0.67	small	possibly	0.197
		PRE-+3 h	-18	24	-0.08	0.12	trivial	most unlikely	0.222
		POST-+1 h	-56	62	-0.47	0.66	small	possibly	0.164
		POST-+3 h	-13	26	-0.08	0.17	trivial	very unlikely	0.335
**p-UBF^{Ser388}**	RT	PRE-POST	-3	18	-0.05	0.26	trivial	very unlikely	0.881
		PRE-+1 h	72	40	0.76	0.32	moderate	very likely	0.018
		PRE-+3 h	117	67	0.93	0.37	moderate	very likely	0.013
		POST-+1 h	78	58	0.82	0.45	moderate	likely	0.010
		POST-+3 h	125	72	1.15	0.45	moderate	very likely	0.001

Measure	Group	Comparison	Mean difference		Standardised effect size (ES)		Effect magnitude	Qualitative likelihood of true effect magnitude being substantial	*P* value
			% difference	±90% CL	ES (*d*)	±90% CL			
	HIT+RT	PRE-POST	18	20	0.29	0.29	small	unlikely	0.220
		PRE-+1 h	42	25	0.55	0.27	small	likely	0.061
		PRE-+3 h	36	41	0.42	0.40	small	possibly	0.088
		POST-+1 h	20	12	0.32	0.17	small	unlikely	0.022
		POST-+3 h	16	23	0.25	0.34	small	unlikely	0.179
	MICT+RT	PRE-POST	26	34	0.28	0.32	small	unlikely	0.144
		PRE-+1 h	41	39	0.38	0.31	small	possibly	0.079
		PRE-+3 h	2	18	0.02	0.19	trivial	most unlikely	0.825
		POST-+1 h	12	40	0.14	0.42	trivial	unlikely	0.516
		POST-+3 h	-19	14	-0.25	0.21	small	unlikely	0.047
**Total-UBF**	RT	PRE-POST	1	8	0.01	0.15	trivial	most unlikely	0.869
		PRE-+1 h	-7	14	-0.11	0.23	trivial	very unlikely	0.453
		PRE-+3 h	0	15	0.01	0.20	trivial	most unlikely	0.960
		POST-+1 h	-7	19	-0.13	0.35	trivial	very unlikely	0.479
		POST-+3 h	0	22	-0.01	0.39	trivial	very unlikely	0.976
	HIT+RT	PRE-POST	9	17	0.15	0.25	trivial	very unlikely	0.291
		PRE-+1 h	17	14	0.23	0.17	small	unlikely	0.031
		PRE-+3 h	20	22	0.26	0.26	small	unlikely	0.079
		POST-+1 h	7	16	0.11	0.24	trivial	very unlikely	0.426
		POST-+3 h	9	22	0.15	0.32	trivial	unlikely	0.400

Measure	Group	Comparison	Mean difference		Standardised effect size (ES)		Effect magnitude	Qualitative likelihood of true effect magnitude being substantial	*P* value
			% difference	±90% CL	ES (*d*)	±90% CL			
	MICT+RT	PRE-POST	18	14	0.44	0.31	small	possibly	0.023
		PRE-+1 h	8	21	0.20	0.47	small	unlikely	0.449
		PRE-+3 h	21	14	0.46	0.26	small	possibly	0.105
		POST-+1 h	-8	18	-0.23	0.51	small	possibly	0.378
		POST-+3 h	2	13	0.06	0.34	trivial	unlikely	0.803
**Total Cyclin D1**	RT	PRE-POST	-1	11	0.00	0.07	trivial	most unlikely	0.914
		PRE-+1 h	-15	12	-0.11	0.09	trivial	most unlikely	0.039
		PRE-+3 h	-22	17	-0.16	0.14	trivial	very unlikely	0.076
		POST-+1 h	-16	12	-0.11	0.09	trivial	most unlikely	0.047
		POST-+3 h	-22	18	-0.16	0.15	trivial	very unlikely	0.073
	HIT+RT	PRE-POST	-11	14	-0.19	0.24	trivial	unlikely	0.418
		PRE-+1 h	-41	12	-0.85	0.32	moderate	very likely	0.001
		PRE-+3 h	-20	9	-0.35	0.19	small	possibly	0.110
		POST-+1 h	-34	7	-0.66	0.16	moderate	very likely	0.008
		POST-+3 h	-10	13	-0.16	0.23	trivial	very unlikely	0.178
	MICT+RT	PRE-POST	-17	8	-0.19	0.10	trivial	most unlikely	0.064
		PRE-+1 h	-31	21	-0.38	0.31	small	possibly	0.092
		PRE-+3 h	-2	32	-0.02	0.32	trivial	very unlikely	0.935
		POST-+1 h	-17	28	-0.19	0.34	trivial	unlikely	0.293
		POST-+3 h	18	41	0.17	0.35	trivial	unlikely	0.398

mRNA target	Group	Comparison	Mean difference		Standardised effect size (ES)		Effect magnitude	Qualitative likelihood of true effect magnitude being substantial	*P* value
			% difference	±90% CL	ES (*d*)	±90% CL			
**TIF-1A mRNA**	RT	PRE-POST	-6	14	-0.14	0.33	trivial	unlikely	0.433
		POST-+3 h	26	12	0.53	0.21	small	likely	0.003
		PRE-+3 h	19	18	0.39	0.33	small	possibly	0.086
	HIT+RT	PRE-POST	-8	12	-0.11	0.17	trivial	very unlikely	0.279
		POST-+3 h	5	33	0.07	0.41	trivial	unlikely	0.753
		PRE-+3 h	-3	33	-0.04	0.44	trivial	very unlikely	0.860
	MICT+RT	PRE-POST	-8	11	-0.16	0.24	trivial	unlikely	0.214
		POST-+3 h	36	35	0.59	0.50	small	likely	0.038
		PRE-+3 h	25	21	0.43	0.32	small	possibly	0.028
**UBF mRNA**	RT	PRE-POST	-14	13	-0.30	0.30	small	possibly	0.100
		POST-+3 h	-20	28	-0.45	0.69	small	possibly	0.218
		PRE-+3 h	-31	18	-0.75	0.51	moderate	likely	0.025
	HIT+RT	PRE-POST	8	8	0.14	0.15	trivial	very unlikely	0.421
		POST-+3 h	-9	17	-0.17	0.35	trivial	unlikely	0.386
		PRE-+3 h	-2	19	-0.04	0.35	trivial	very unlikely	0.837
	MICT+RT	PRE-POST	4	15	0.10	0.32	trivial	unlikely	0.533
		POST-+3 h	-11	17	-0.27	0.44	small	possibly	0.361
		PRE-+3 h	-7	19	-0.17	0.46	trivial	unlikely	0.599

mRNA target	Group	Comparison	Mean difference		Standardised effect size (ES)		Effect magnitude	Qualitative likelihood of true effect magnitude being substantial	*P* value
			% difference	±90% CL	ES (*d*)	±90% CL			
**POLR1B mRNA**	RT	PRE-POST	-26	16	-0.44	0.32	small	possibly	0.026
		POST-+3 h	8	32	0.11	0.42	trivial	very unlikely	0.622
		PRE-+3 h	-20	19	-0.33	0.35	small	possibly	0.087
	HIT+RT	PRE-POST	1	10	0.02	0.15	trivial	most unlikely	0.810
		POST-+3 h	44	42	0.57	0.44	small	likely	0.047
		PRE-+3 h	46	38	0.59	0.40	small	likely	0.023
	MICT+RT	PRE-POST	-5	20	-0.06	0.27	trivial	very unlikely	0.634
		POST-+3 h	48	43	0.51	0.37	small	possibly	0.033
		PRE-+3 h	41	33	0.45	0.30	small	possibly	0.025
**Cyclin D1 mRNA**	RT	PRE-POST	51	45	0.29	0.21	small	unlikely	0.424
		POST-+3 h	10	41	0.07	0.26	trivial	very unlikely	0.603
		PRE-+3 h	66	68	0.36	0.28	small	possibly	0.364
	HIT+RT	PRE-POST	101	54	0.59	0.22	small	likely	0.001
		POST-+3 h	10	43	0.08	0.32	trivial	unlikely	0.648
		PRE-+3 h	121	115	0.66	0.42	moderate	likely	0.014
	MICT+RT	PRE-POST	36	67	0.29	0.44	small	possibly	0.292
		POST-+3 h	4	44	0.04	0.38	trivial	unlikely	0.833
		PRE-+3 h	42	63	0.33	0.41	small	possibly	0.217

rRNA target	Group	Comparison	Mean difference		Standardised effect size (ES)		Effect magnitude	Qualitative likelihood of true effect magnitude being substantial	*P* value
			% difference	±90% CL	ES (*d*)	±90% CL			
**Total RNA**	RT	PRE-POST	-11	5	-0.17	0.09	trivial	most unlikely	0.025
	HIT+RT	PRE-POST	32	18	0.12	0.08	trivial	unlikely	0.083
	MICT+RT	PRE-POST	20	15	0.30	0.15	small	most unlikely	0.077
**45S pre-rRNA**	RT	PRE-POST	-32	22	-0.40	0.33	small	possibly	0.138
		POST-+3 h	12	42	0.12	0.39	trivial	very unlikely	0.539
		PRE-+3 h	-23	34	-0.27	0.45	small	possibly	0.314
	HIT+RT	PRE-POST	8	26	0.07	0.21	trivial	very unlikely	0.514
		POST-+3 h	69	107	0.48	0.54	small	possibly	0.110
		PRE-+3 h	83	116	0.55	0.54	small	possibly	0.071
	MICT+RT	PRE-POST	20	24	0.21	0.22	small	unlikely	0.099
		POST-+3 h	43	62	0.40	0.47	small	possibly	0.121
		PRE-+3 h	71	80	0.61	0.51	moderate	likely	0.041
**5.8S rRNA**	RT	PRE-POST	-51	16	-0.69	0.31	moderate	likely	0.017
		POST-+3 h	22	63	0.20	0.48	small	unlikely	0.421
		PRE-+3 h	-40	29	-0.49	0.44	small	possibly	0.099
	HIT+RT	PRE-POST	10	20	0.08	0.15	trivial	most unlikely	0.469
		POST-+3 h	39	87	0.27	0.47	small	possibly	0.280

rRNA target	Group	Comparison	Mean difference		Standardised effect size (ES)		Effect magnitude	Qualitative likelihood of true effect magnitude being substantial	*P* value
			% difference	±90% CL	ES (*d*)	±90% CL			
		PRE-+3 h	54	108	0.35	0.52	small	possibly	0.244
	MICT+RT	PRE-POST	8	36	0.05	0.24	trivial	very unlikely	0.632
		POST-+3 h	46	73	0.27	0.35	small	unlikely	0.200
		PRE-+3 h	57	97	0.33	0.42	small	possibly	0.194
**5.8S rRNA (span)**	RT	PRE-POST	-36	15	-0.51	0.27	small	likely	0.027
		POST-+3 h	2	26	0.02	0.29	trivial	very unlikely	0.923
		PRE-+3 h	-35	19	-0.49	0.33	small	possibly	0.017
	HIT+RT	PRE-POST	35	35	0.29	0.24	small	unlikely	0.244
		POST-+3 h	54	74	0.41	0.44	small	possibly	0.127
		PRE-+3 h	95	100	0.63	0.47	moderate	likely	0.033
	MICT+RT	PRE-POST	-1	47	-0.01	0.47	trivial	unlikely	0.962
		POST-+3 h	29	61	0.26	0.46	small	possibly	0.269
		PRE-+3 h	27	59	0.25	0.45	small	possibly	0.330
**18S rRNA**	RT	PRE-POST	112	59	0.34	0.12	small	unlikely	0.509
		POST-+3 h	-8	41	-0.04	0.20	trivial	very unlikely	0.732
		PRE-+3 h	96	40	0.30	0.09	small	very unlikely	0.525
	HIT+RT	PRE-POST	3	13	0.04	0.17	trivial	most unlikely	0.762
		POST-+3 h	28	54	0.34	0.55	small	possibly	0.243
		PRE-+3 h	33	44	0.38	0.44	small	possibly	0.142

rRNA target	Group	Comparison	Mean difference		Standardised effect size (ES)		Effect magnitude	Qualitative likelihood of true effect magnitude being substantial	*P* value
			% difference	±90% CL	ES (*d*)	±90% CL			
	MICT+RT	PRE-POST	-22	17	-0.30	0.26	small	unlikely	0.429
		POST-+3 h	-9	37	-0.11	0.47	trivial	unlikely	0.635
		PRE-+3 h	-29	19	-0.41	0.31	small	possibly	0.270
**18S rRNA (span)**	RT	PRE-POST	-35	29	-0.30	0.30	small	possibly	0.078
		POST-+3 h	29	90	0.18	0.45	trivial	unlikely	0.446
		PRE-+3 h	-16	40	-0.12	0.32	trivial	unlikely	0.465
	HIT+RT	PRE-POST	28	85	0.11	0.29	trivial	unlikely	0.509
		POST-+3 h	49	106	0.18	0.30	trivial	unlikely	0.255
		PRE-+3 h	90	186	0.30	0.40	small	possibly	0.222
	MICT+RT	PRE-POST	4	22	0.02	0.09	trivial	most unlikely	0.773
		POST-+3 h	63	48	0.21	0.12	small	very unlikely	0.029
		PRE-+3 h	69	75	0.22	0.18	small	unlikely	0.106
**28S rRNA**	RT	PRE-POST	-33	15	-0.49	0.28	small	possibly	0.037
		POST-+3 h	3	33	0.03	0.39	trivial	unlikely	0.867
		PRE-+3 h	-31	22	-0.46	0.39	small	possibly	0.052
	HIT+RT	PRE-POST	16	14	0.16	0.13	trivial	most unlikely	0.097
		POST-+3 h	17	59	0.16	0.51	trivial	unlikely	0.530
		PRE-+3 h	36	76	0.32	0.56	small	possibly	0.287

rRNA target	Group	Comparison	Mean difference		Standardised effect size (ES)		Effect magnitude	Qualitative likelihood of true effect magnitude being substantial	*P* value
			% difference	±90% CL	ES (*d*)	±90% CL			
	MICT+RT	PRE-POST	10	20	0.13	0.25	trivial	very unlikely	0.380
		POST-+3 h	4	38	0.05	0.49	trivial	unlikely	0.826
		PRE-+3 h	14	46	0.18	0.53	trivial	unlikely	0.525
**28S rRNA (span)**	RT	PRE-POST	-19	21	-0.22	0.27	small	unlikely	0.163
		POST-+3 h	1	56	0.01	0.55	trivial	unlikely	0.963
		PRE-+3 h	-18	34	-0.20	0.42	small	unlikely	0.362
	HIT+RT	PRE-POST	27	40	0.26	0.32	small	unlikely	0.251
		POST-+3 h	-21	26	-0.24	0.34	small	unlikely	0.186
		PRE-+3 h	1	43	0.01	0.44	trivial	unlikely	0.970
	MICT+RT	PRE-POST	-15	15	-0.20	0.21	small	unlikely	0.229
		POST-+3 h	44	58	0.44	0.48	small	possibly	0.093
		PRE-+3 h	22	59	0.24	0.56	small	possibly	0.394

Measure	Group	Comparison	Mean difference	Standardised effect size (ES)	Effect magnitude	Qualitative likelihood of true [illegible]	*P* value

			% difference	±90% CL	ES (*d*)	±90% CL			
**Type I fibre CSA**	RT	PRE-POST	15	13	0.10	0.08	trivial	most unlikely	0.035
	HIT+RT	PRE-POST	-23	19	-0.09	0.08	trivial	most unlikely	0.135
	MICT+RT	PRE-POST	0.4	17	0.00	-0.14	trivial	most unlikely	0.989
**Type II fibre CSA**	RT	PRE-POST	19	27	0.09	0.12	trivial	most unlikely	0.139
	HIT+RT	PRE-POST	0.4	24	0.00	0.08	trivial	most unlikely	0.974
	MICT+RT	PRE-POST	16	14	0.19	0.16	trivial	very likely	0.344
Maximal strength									
**1-RM leg press**	RT	PRE-POST	38.5	8.5	1.26	0.24	large	most likely	<0.001
	HIT+RT	PRE-POST	28.7	5.3	1.17	0.19	moderate	most likely	<0.001
	MICT+RT	PRE-POST	27.5	4.6	0.81	0.12	moderate	most likely	<0.001
Body composition									
**Lower-body lean mass**	RT	PRE-POST	4.1	2.0	0.33	0.16	small	likely	0.023
	MICT+RT	PRE-POST	3.6	2.4	0.45	0.30	small	likely	0.052

Supplementary data Table 2. Summary of magnitude-based inference (MBI) data for all between-group comparisons.

Protein target	Comparison	Change between	Mean difference in change		Standardised effect size (ES)		Effect magnitude	Qualitative likelihood of true effect magnitude being substantial
			% difference	±90% CL	ES (*d*)	±90% CL		
**p-mTOR^{Ser2448}**	HIT+RT vs. RT	PRE-POST	7	71	0.07	0.60	trivial	unlikely
		PRE-+1 h	-37	45	-0.34	0.49	small	possibly
		PRE-+3 h	-46	41	-0.48	0.54	small	possibly
		POST-+1 h	-36	47	-0.44	0.66	small	possibly
		POST-+3 h	-29	70	-0.33	0.84	small	possibly
	MICT+RT vs. RT	PRE-POST	-26	68	-0.29	0.80	small	possibly
		PRE-+1 h	-15	88	-0.11	0.62	trivial	unlikely
		PRE-+3 h	-64	36	-0.64	0.55	moderate	likely
		POST-+1 h	-14	90	-0.15	0.89	trivial	possibly
		POST-+3 h	-36	61	-0.44	0.83	small	possibly
	HIT+RT vs. MICT+RT	PRE-POST	-31	53	-0.46	0.88	small	possibly
		PRE-+1 h	24	118	0.18	0.70	trivial	possibly
		PRE-+3 h	-53	43	-0.54	0.58	small	possibly
		POST-+1 h	35	136	0.38	1.11	small	possibly
		POST-+3 h	-10	41	-0.13	0.56	trivial	unlikely
**Total mTOR**	HIT+RT vs. RT	PRE-POST	1	30	0.03	0.61	trivial	unlikely
		PRE-+1 h	-27	21	-0.51	0.47	small	possibly
		PRE-+3 h	-25	32	-0.36	0.51	small	possibly
		POST-+1 h	-23	20	-0.55	0.54	small	possibly

Protein target	Comparison	Change between	Mean difference in change		Standardised effect size (ES)		Effect magnitude	Qualitative likelihood of true effect magnitude being substantial
			% difference	±90% CL	ES (*d*)	±90% CL		
		POST-+3 h	-31	26	-0.77	0.76	moderate	likely
	MICT+RT vs. RT	PRE-POST	23	44	0.31	0.54	small	possibly
		PRE-+1 h	-25	28	-0.35	0.46	small	possibly
		PRE-+3 h	-27	37	-0.38	0.59	small	possibly
		POST-+1 h	-21	28	-0.36	0.54	small	possibly
		POST-+3 h	-31	30	-0.57	0.65	small	possibly
	HIT+RT vs. MICT+RT	PRE-POST	21	35	0.43	0.64	small	possibly
		PRE-+1 h	-3	34	-0.05	0.55	trivial	unlikely
		PRE-+3 h	4	47	0.06	0.61	trivial	unlikely
		POST-+1 h	3	35	0.07	0.75	trivial	unlikely
		POST-+3 h	1	41	0.01	0.89	trivial	unlikely
**p-p70S6K1^{Thr389}**	HIT+RT vs. RT	PRE-POST	33	97	0.38	0.91	small	possibly
		PRE-+1 h	-58	22	-0.98	0.56	moderate	very likely
		PRE-+3 h	-67	30	-1.02	0.75	moderate	likely
		POST-+1 h	-38	36	-0.65	0.74	moderate	possibly
		POST-+3 h	-47	50	-0.86	1.13	moderate	likely
	MICT+RT vs. RT	PRE-POST	-13	60	-0.17	0.82	trivial	possibly
		PRE-+1 h	-50	27	-0.89	0.67	moderate	likely
		PRE-+3 h	-74	20	-1.53	0.80	large	very likely
		POST-+1 h	-27	44	-0.40	0.73	small	possibly
		POST-+3 h	-50	46	-0.88	1.05	moderate	likely

Protein target	Comparison	Change between	Mean difference in change		Standardised effect size (ES)		Effect magnitude	Qualitative likelihood of true effect magnitude being substantial
			% difference	±90% CL	ES (*d*)	±90% CL		
	HIT+RT vs. MICT+RT	PRE-POST	-34	37	-0.65	0.83	moderate	possibly
		PRE-+1 h	-39	51	-0.65	1.00	moderate	possibly
		PRE-+3 h	-63	24	-1.16	0.72	moderate	very likely
		POST-+1 h	19	77	0.27	0.94	small	possibly
		POST-+3 h	-5	44	-0.08	0.69	trivial	unlikely
**Total-p70S6K1**	HIT+RT vs. RT	PRE-POST	7	19	0.12	0.31	trivial	unlikely
		PRE-+1 h	-6	16	-0.08	0.23	trivial	very unlikely
		PRE-+3 h	18	38	0.20	0.38	small	unlikely
		POST-+1 h	-3	19	-0.05	0.34	trivial	very unlikely
		POST-+3 h	3	31	0.05	0.52	trivial	unlikely
	MICT+RT vs. RT	PRE-POST	-22	38	-0.26	0.50	small	possibly
		PRE-+1 h	25	44	0.20	0.30	small	unlikely
		PRE-+3 h	41	52	0.35	0.38	small	possibly
		POST-+1 h	29	46	0.27	0.36	small	possibly
		POST-+3 h	64	93	0.52	0.57	small	possibly
	HIT+RT vs. MICT+RT	PRE-POST	-27	36	-0.47	0.71	small	possibly
		PRE-+1 h	21	44	0.25	0.46	small	possibly
		PRE-+3 h	28	34	0.42	0.45	small	possibly
		POST-+1 h	33	45	0.43	0.50	small	possibly
		POST-+3 h	59	86	0.70	0.78	moderate	likely

Protein target	Comparison	Change between	Mean difference in change		Standardised effect size (ES)		Effect magnitude	Qualitative likelihood of true effect magnitude being substantial
			% difference	±90% CL	ES (*d*)	±90% CL		
**p-rps6$^{Ser235/236}$**	HIT+RT vs. RT	PRE-POST	7	99	0.04	0.48	trivial	unlikely
		PRE-+1 h	-34	86	-0.15	0.40	small	unlikely
		PRE-+3 h	-94	8	-0.95	0.39	moderate	very likely
		POST-+1 h	-28	85	-0.19	0.58	small	possibly
		POST-+3 h	-63	41	-0.57	0.56	small	possibly
	MICT+RT vs. RT	PRE-POST	-1	91	-0.01	0.44	trivial	unlikely
		PRE-+1 h	-17	84	-0.07	0.35	trivial	unlikely
		PRE-+3 h	-97	4	-1.30	0.42	large	most likely
		POST-+1 h	-10	79	-0.06	0.42	trivial	unlikely
		POST-+3 h	-74	29	-0.72	0.51	moderate	likely
	HIT+RT vs. MICT+RT	PRE-POST	-8	46	-0.06	0.38	trivial	unlikely
		PRE-+1 h	8	129	0.03	0.46	trivial	unlikely
		PRE-+3 h	-92	10	-1.01	0.41	moderate	very likely
		POST-+1 h	25	128	0.18	0.72	trivial	possibly
		POST-+3 h	-30	70	-0.29	0.70	small	possibly
**Total-rps6**	HIT+RT vs. RT	PRE-POST	13	24	0.09	0.15	trivial	most unlikely
		PRE-+1 h	-12	23	-0.07	0.14	trivial	most unlikely
		PRE-+3 h	-6	24	-0.03	0.13	trivial	most unlikely
		POST-+1 h	-14	16	-0.11	0.13	trivial	most unlikely
		POST-+3 h	-7	20	-0.05	0.15	trivial	most unlikely

Protein target	Comparison	Change between	Mean difference in change		Standardised effect size (ES)		Effect magnitude	Qualitative likelihood of true effect magnitude being substantial
			% difference	±90% CL	ES (*d*)	±90% CL		
	MICT+RT vs. RT	PRE-POST	18	29	0.11	0.15	trivial	most unlikely
		PRE-+1 h	1	31	0.00	0.15	trivial	most unlikely
		PRE-+3 h	-17	23	-0.08	0.12	trivial	most unlikely
		POST-+1 h	-1	25	-0.01	0.16	trivial	most unlikely
		POST-+3 h	-6	26	-0.04	0.17	trivial	most unlikely
	HIT+RT vs. MICT+RT	PRE-POST	5	26	0.03	0.17	trivial	most unlikely
		PRE-+1 h	3	26	0.02	0.14	trivial	most unlikely
		PRE-+3 h	-21	22	-0.11	0.13	trivial	most unlikely
		POST-+1 h	15	26	0.09	0.15	trivial	most unlikely
		POST-+3 h	1	25	0.01	0.17	trivial	most unlikely
**p-4E-BP1$^{Thr37/46}$**	HIT+RT vs. RT	PRE-POST	-31	34	-0.52	0.67	moderate	possibly
		PRE-+1 h	-20	49	-0.24	0.61	small	possibly
		PRE-+3 h	-3	57	-0.04	0.59	trivial	unlikely
		POST-+1 h	8	32	0.11	0.41	trivial	unlikely
		POST-+3 h	32	45	0.38	0.47	small	possibly
	MICT+RT vs. RT	PRE-POST	-10	48	-0.19	0.90	trivial	possibly
		PRE-+1 h	-14	52	-0.20	0.77	small	possibly
		PRE-+3 h	-1	63	-0.01	0.77	trivial	unlikely
		POST-+1 h	17	32	0.27	0.47	small	possibly
		POST-+3 h	46	66	0.66	0.76	moderate	possibly

Protein target	Comparison	Change between	Mean difference in change		Standardised effect size (ES)		Effect magnitude	Qualitative likelihood of true effect magnitude being substantial
			% difference	±90% CL	ES (*d*)	±90% CL		
	HIT+RT vs. MICT+RT	PRE-POST	30	57	0.53	0.87	small	possibly
		PRE-+1 h	16	38	0.22	0.48	small	possibly
		PRE-+3 h	9	47	0.11	0.53	trivial	unlikely
		POST-+1 h	8	32	0.16	0.60	trivial	unlikely
		POST-+3 h	11	52	0.22	0.93	small	possibly
**Total-4E-BP1**	HIT+RT vs. RT	PRE-POST	17	29	0.27	0.42	small	possibly
		PRE-+1 h	23	78	0.19	0.53	trivial	unlikely
		PRE-+3 h	35	120	0.19	0.50	trivial	unlikely
		POST-+1 h	19	57	0.30	0.79	small	possibly
		POST-+3 h	29	91	0.44	1.12	small	possibly
	MICT+RT vs. RT	PRE-POST	12	24	0.22	0.42	small	unlikely
		PRE-+1 h	27	83	0.23	0.59	small	possibly
		PRE-+3 h	19	109	0.12	0.54	trivial	unlikely
		POST-+1 h	22	63	0.40	0.98	small	possibly
		POST-+3 h	17	83	0.32	1.30	small	possibly
	HIT+RT vs. MICT+RT	PRE-POST	-4	18	-0.08	0.33	trivial	unlikely
		PRE-+1 h	-9	27	-0.14	0.43	trivial	unlikely
		PRE-+3 h	-21	25	-0.32	0.42	small	possibly
		POST-+1 h	3	27	0.05	0.45	trivial	unlikely
		POST-+3 h	-9	12	-0.17	0.24	trivial	unlikely

Protein target	Comparison	Change between	Mean difference in change		Standardised effect size (ES)		Effect magnitude	Qualitative likelihood of true effect magnitude being substantial
			% difference	±90% CL	ES (*d*)	±90% CL		
**p-ACC^{Ser79}**	HIT+RT vs. RT	PRE-POST	-23	40	-0.24	0.47	small	possibly
		PRE-+1 h	35	103	0.21	0.49	small	unlikely
		PRE-+3 h	44	115	0.22	0.44	small	unlikely
		POST-+1 h	99	100	0.65	0.46	moderate	likely
		POST-+3 h	169	168	0.94	0.56	moderate	likely
	MICT+RT vs. RT	PRE-POST	-22	41	-0.23	0.47	small	possibly
		PRE-+1 h	-43	42	-0.39	0.48	small	possibly
		PRE-+3 h	75	106	0.40	0.40	small	possibly
		POST-+1 h	-16	38	-0.16	0.41	trivial	unlikely
		POST-+3 h	39	87	0.30	0.55	small	possibly
	HIT+RT vs. MICT+RT	PRE-POST	1	37	0.01	0.34	trivial	very unlikely
		PRE-+1 h	-63	29	-0.82	0.59	moderate	likely
		PRE-+3 h	-16	62	-0.14	0.56	trivial	unlikely
		POST-+1 h	-58	23	-0.82	0.49	moderate	likely
		POST-+3 h	-48	39	-0.63	0.67	moderate	possibly
**p-AMPK^{Thr172}**	HIT+RT vs. RT	PRE-POST	-18	89	-0.18	0.84	trivial	possibly
		PRE-+1 h	-51	47	-0.49	0.58	small	possibly
		PRE-+3 h	-68	39	-0.73	0.66	moderate	likely
		POST-+1 h	-37	52	-0.41	0.68	small	possibly
		POST-+3 h	-54	49	-0.69	0.83	moderate	possibly

Protein target	Comparison	Change between	Mean difference in change		Standardised effect size (ES)		Effect magnitude	Qualitative likelihood of true effect magnitude being substantial
			% difference	±90% CL	ES (*d*)	±90% CL		
	MICT+RT vs. RT	PRE-POST	10	110	0.09	0.79	trivial	unlikely
		PRE-+1 h	-44	40	-0.45	0.51	small	possibly
		PRE-+3 h	-75	26	-1.02	0.67	moderate	likely
		POST-+1 h	-28	37	-0.30	0.45	small	possibly
		POST-+3 h	-59	44	-0.79	0.83	moderate	likely
	HIT+RT vs. MICT+RT	PRE-POST	34	89	0.28	0.60	small	possibly
		PRE-+1 h	7	71	0.06	0.49	trivial	unlikely
		PRE-+3 h	-34	41	-0.34	0.48	small	possibly
		POST-+1 h	13	90	0.12	0.70	trivial	unlikely
		POST-+3 h	-11	33	-0.11	0.35	trivial	unlikely
**Total AMPK**	HIT+RT vs. RT	PRE-POST	22	33	0.53	0.74	small	possibly
		PRE-+1 h	3	16	0.06	0.31	trivial	very unlikely
		PRE-+3 h	-23	15	-0.46	0.35	small	possibly
		POST-+1 h	12	17	0.30	0.41	small	possibly
		POST-+3 h	-9	14	-0.26	0.42	small	possibly
	MICT+RT vs. RT	PRE-POST	4	19	0.13	0.57	trivial	unlikely
		PRE-+1 h	-15	14	-0.42	0.43	small	possibly
		PRE-+3 h	4	18	0.08	0.38	trivial	unlikely
		POST-+1 h	-8	15	-0.25	0.50	small	possibly
		POST-+3 h	1	15	0.03	0.47	trivial	unlikely

Protein target	Comparison	Change between	Mean difference in change		Standardised effect size (ES)		Effect magnitude	Qualitative likelihood of true effect magnitude being substantial
			% difference	±90% CL	ES (*d*)	±90% CL		
	HIT+RT vs. MICT+RT	PRE-POST	-14	26	-0.51	0.99	small	possibly
		PRE-+1 h	-37	17	-1.18	0.69	moderate	very likely
		PRE-+3 h	-6	33	-0.13	0.70	trivial	unlikely
		POST-+1 h	-17	15	-0.63	0.60	moderate	possibly
		POST-+3 h	11	17	0.35	0.51	small	possibly
**p-TIF-1A^{Ser649}**	HIT+RT vs. RT	PRE-POST	74	171	0.58	0.91	small	possibly
		PRE-+1 h	-55	32	-0.74	0.61	moderate	likely
		PRE-+3 h	-73	39	-0.80	0.71	moderate	likely
		POST-+1 h	-40	35	-0.54	0.57	small	possibly
		POST-+3 h	-52	46	-0.76	0.89	moderate	likely
	MICT+RT vs. RT	PRE-POST	0	82	0.00	0.71	trivial	unlikely
		PRE-+1 h	-56	36	-0.68	0.63	moderate	likely
		PRE-+3 h	-86	16	-1.21	0.59	large	very likely
		POST-+1 h	-41	43	-0.51	0.65	small	possibly
		POST-+3 h	-75	24	-1.31	0.80	large	very likely
	HIT+RT vs. MICT+RT	PRE-POST	-42	48	-0.59	0.82	moderate	possibly
		PRE-+1 h	-58	47	-0.90	1.01	moderate	likely
		PRE-+3 h	-83	15	-1.34	0.60	large	very likely
		POST-+1 h	-2	80	-0.02	0.81	trivial	unlikely
		POST-+3 h	-47	36	-0.69	0.70	moderate	likely

Protein target	Comparison	Change between	Mean difference in change		Standardised effect size (ES)		Effect magnitude	Qualitative likelihood of true effect magnitude being substantial
			% difference	±90% CL	ES (*d*)	±90% CL		
**Total-TIF-1A**	HIT+RT vs. RT	PRE-POST	-14	17	-0.37	0.47	small	possibly
		PRE-+1 h	26	52	0.35	0.59	small	possibly
		PRE-+3 h	32	75	0.26	0.52	small	possibly
		POST-+1 h	24	41	0.52	0.78	small	possibly
		POST-+3 h	17	69	0.39	1.34	small	possibly
	MICT+RT vs. RT	PRE-POST	-4	28	-0.09	0.64	trivial	unlikely
		PRE-+1 h	-39	76	-0.32	0.68	small	possibly
		PRE-+3 h	247	431	0.77	0.64	moderate	likely
		POST-+1 h	-40	75	-1.15	2.36	moderate	possibly
		POST-+3 h	49	56	0.90	0.83	moderate	likely
	HIT+RT vs. MICT+RT	PRE-POST	12	31	0.26	0.62	small	possibly
		PRE-+1 h	-43	71	-0.35	0.65	small	possibly
		PRE-+3 h	245	439	0.70	0.60	moderate	likely
		POST-+1 h	-52	60	-1.65	2.38	large	likely
		POST-+3 h	27	73	0.54	1.24	small	possibly
**p-UBF^{Ser388}**	HIT+RT vs. RT	PRE-POST	22	58	0.28	0.64	small	possibly
		PRE-+1 h	-30	25	-0.46	0.45	small	possibly
		PRE-+3 h	-56	22	-1.06	0.62	moderate	very likely
		POST-+1 h	-32	23	-0.54	0.46	small	possibly
		POST-+3 h	-49	17	-0.92	0.45	moderate	very likely

Protein target	Comparison	Change between	Mean difference in change		Standardised effect size (ES)		Effect magnitude	Qualitative likelihood of true effect magnitude being substantial
			% difference	±90% CL	ES (*d*)	±90% CL		
	MICT+RT vs. RT	PRE-POST	30	63	0.35	0.62	small	possibly
		PRE-+1 h	-35	29	-0.53	0.53	small	possibly
		PRE-+3 h	-67	17	-1.40	0.62	large	very likely
		POST-+1 h	-37	27	-0.61	0.55	moderate	possibly
		POST-+3 h	-64	12	-1.35	0.42	large	most likely
	HIT+RT vs. MICT+RT	PRE-POST	7	36	0.14	0.69	trivial	possibly
		PRE-+1 h	-21	33	-0.42	0.72	small	possibly
		PRE-+3 h	-47	19	-0.84	0.48	moderate	likely
		POST-+1 h	-7	31	-0.15	0.70	trivial	possibly
		POST-+3 h	-30	16	-0.74	0.48	moderate	likely
**Total-UBF**	HIT+RT vs. RT	PRE-POST	9	18	0.21	0.41	small	unlikely
		PRE-+1 h	14	24	0.25	0.38	small	unlikely
		PRE-+3 h	2	21	0.03	0.34	trivial	very unlikely
		POST-+1 h	15	26	0.36	0.57	small	possibly
		POST-+3 h	10	29	0.23	0.65	small	possibly
	MICT+RT vs. RT	PRE-POST	18	15	0.51	0.39	small	possibly
		PRE-+1 h	-2	22	-0.04	0.44	trivial	unlikely
		PRE-+3 h	11	26	0.19	0.41	trivial	unlikely
		POST-+1 h	-1	24	-0.04	0.76	trivial	unlikely
		POST-+3 h	3	26	0.08	0.78	trivial	unlikely

Protein target	Comparison	Change between	Mean difference in change		Standardised effect size (ES)		Effect magnitude	Qualitative likelihood of true effect magnitude being substantial
			% difference	±90% CL	ES (*d*)	±90% CL		
	HIT+RT vs. MICT+RT	PRE-POST	8	19	0.18	0.38	trivial	unlikely
		PRE-+1 h	-22	16	-0.46	0.37	small	possibly
		PRE-+3 h	-7	23	-0.13	0.45	trivial	unlikely
		POST-+1 h	-14	19	-0.35	0.49	small	possibly
		POST-+3 h	-6	23	-0.15	0.54	trivial	unlikely
**Total Cyclin D1**	HIT+RT vs. RT	PRE-POST	-11	25	-0.11	0.26	trivial	very unlikely
		PRE-+1 h	-21	19	-0.16	0.16	trivial	very unlikely
		PRE-+3 h	75	55	0.36	0.20	small	possibly
		POST-+1 h	-22	19	-0.23	0.23	small	unlikely
		POST-+3 h	15	28	0.14	0.23	trivial	very unlikely
	MICT+RT vs. RT	PRE-POST	-16	15	-0.18	0.18	trivial	very unlikely
		PRE-+1 h	-1	32	-0.01	0.25	trivial	very unlikely
		PRE-+3 h	82	57	0.47	0.24	small	possibly
		POST-+1 h	-2	32	-0.02	0.33	trivial	very unlikely
		POST-+3 h	51	61	0.42	0.40	small	possibly
	HIT+RT vs. MICT+RT	PRE-POST	-7	27	-0.09	0.39	trivial	unlikely
		PRE-+1 h	41	48	0.34	0.33	small	possibly
		PRE-+3 h	77	56	0.50	0.27	small	possibly
		POST-+1 h	25	45	0.31	0.47	small	possibly
		POST-+3 h	31	49	0.36	0.50	small	possibly

mRNA target	Comparison	Change between	Mean difference in change		Standardised effect size (ES)		Effect magnitude	Qualitative likelihood of true effect magnitude being substantial
			% difference	±90% CL	ES (*d*)	±90% CL		
**TIF-1A mRNA**	HIT+RT vs. RT	PRE-POST	-2	18	-0.06	0.55	trivial	unlikely
		POST-+3 h	-17	26	-0.57	0.93	small	possibly
		PRE-+3 h	-18	27	-0.62	1.01	moderate	possibly
	MICT+RT vs. RT	PRE-POST	-2	17	-0.07	0.53	trivial	unlikely
		POST-+3 h	7	25	0.22	0.71	small	possibly
		PRE-+3 h	5	21	0.15	0.63	trivial	unlikely
	HIT+RT vs. MICT+RT	PRE-POST	0	16	-0.02	0.51	trivial	unlikely
		POST-+3 h	29	45	0.80	1.06	moderate	possibly
		PRE-+3 h	28	42	0.78	1.01	moderate	possibly
**Cyclin D1 mRNA**	HIT+RT vs. RT	PRE-POST	33	146	0.28	0.95	small	possibly
		POST-+3 h	0	49	0.00	0.47	trivial	unlikely
		PRE-+3 h	33	168	0.28	1.06	small	possibly
	MICT+RT vs. RT	PRE-POST	-10	107	-0.10	0.94	trivial	possibly
		POST-+3 h	-5	45	-0.05	0.43	trivial	unlikely
		PRE-+3 h	-15	108	-0.15	0.99	trivial	possibly
	HIT+RT vs. MICT+RT	PRE-POST	-32	39	-0.50	0.71	small	possibly
		POST-+3 h	-5	48	-0.07	0.63	trivial	unlikely
		PRE-+3 h	-36	43	-0.57	0.81	small	possibly

mRNA target	Comparison	Change between	Mean difference in change		Standardised effect size (ES)		Effect magnitude	Qualitative likelihood of true effect magnitude being substantial
			% difference	±90% CL	ES (*d*)	±90% CL		
**POLR1B mRNA**	HIT+RT vs. RT	PRE-POST	37	30	0.87	0.60	moderate	likely
		POST-+3 h	34	51	0.81	1.03	moderate	likely
		PRE-+3 h	83	57	1.69	0.86	large	very likely
	MICT+RT vs. RT	PRE-POST	29	33	0.51	0.51	small	possibly
		POST-+3 h	38	50	0.64	0.72	moderate	possibly
		PRE-+3 h	77	52	1.14	0.58	moderate	very likely
	HIT+RT vs. MICT+RT	PRE-POST	-6	19	-0.15	0.48	trivial	unlikely
		POST-+3 h	3	39	0.06	0.90	trivial	possibly
		PRE-+3 h	-3	31	-0.08	0.76	trivial	unlikely

rRNA target	Comparison	Change between	Mean difference in change		Standardised effect size (ES)		Effect magnitude	Qualitative likelihood of true effect magnitude being substantial
			% difference	±90% CL	ES (*d*)	±90% CL		
**Total RNA**	HIT+RT vs. RT	PRE-POST	48	39	1.14	0.76	moderate	very likely
	MICT+RT vs. RT	PRE-POST	34	24	1.24	0.75	large	very likely
	HIT+RT vs. MICT+RT	PRE-POST	-9	26	-0.34	0.99	small	possibly
**45S pre-rRNA**	HIT+RT vs. RT	PRE-POST	58	76	0.71	0.71	moderate	likely
		POST-+3 h	50	97	0.63	0.94	moderate	possibly
		PRE-+3 h	138	169	1.34	1.02	large	likely
	MICT+RT vs. RT	PRE-POST	75	81	0.85	0.68	moderate	likely
		POST-+3 h	27	63	0.36	0.72	small	possibly
		PRE-+3 h	123	133	1.21	0.86	large	likely
	HIT+RT vs. MICT+RT	PRE-POST	11	30	0.18	0.48	trivial	possibly
		POST-+3 h	-16	56	-0.30	1.11	small	possibly
		PRE-+3 h	-6	63	-0.12	1.13	trivial	possibly
**5.8S rRNA**	HIT+RT vs. RT	PRE-POST	125	109	1.27	0.73	large	very likely
		POST-+3 h	14	80	0.20	1.02	small	possibly
		PRE-+3 h	156	215	1.47	1.20	large	likely
	MICT+RT vs. RT	PRE-POST	120	111	0.99	0.61	moderate	likely
		POST-+3 h	19	80	0.22	0.79	small	possibly

rRNA target	Comparison	Change between	Mean difference in change		Standardised effect size (ES)		Effect magnitude	Qualitative likelihood of true effect magnitude being substantial
			% difference	±90% CL	ES (*d*)	±90% CL		
		PRE-+3 h	161	207	1.20	0.91	large	likely
	HIT+RT vs. MICT+RT	PRE-POST	-2	35	-0.03	0.48	trivial	unlikely
		POST-+3 h	4	77	0.06	0.95	trivial	possibly
		PRE-+3 h	2	93	0.03	1.12	trivial	possibly
**5.8S rRNA (span)**	HIT+RT vs. RT	PRE-POST	112	116	1.40	0.97	large	very likely
		POST-+3 h	51	82	0.76	0.96	moderate	possibly
		PRE-+3 h	198	161	2.03	0.96	very large	very likely
	MICT+RT vs. RT	PRE-POST	53	86	0.74	0.92	moderate	possibly
		POST-+3 h	27	61	0.41	0.80	small	possibly
		PRE-+3 h	95	98	1.15	0.84	moderate	likely
	HIT+RT vs. MICT+RT	PRE-POST	-28	48	-0.55	1.05	small	possibly
		POST-+3 h	-16	51	-0.29	0.96	small	possibly
		PRE-+3 h	-35	42	-0.72	1.02	moderate	possibly
**18S rRNA**	HIT+RT vs. RT	PRE-POST	-51	187	-0.45	1.30	small	possibly
		POST-+3 h	39	76	0.21	0.33	small	unlikely
		PRE-+3 h	-32	230	-0.25	1.22	small	possibly
	MICT+RT vs. RT	PRE-POST	-63	145	-0.56	1.17	small	possibly
		POST-+3 h	-1	52	-0.01	0.28	trivial	very unlikely
		PRE-+3 h	-64	124	-0.57	1.09	small	possibly

rRNA target	Comparison	Change between	Mean difference in change		Standardised effect size (ES)		Effect magnitude	Qualitative likelihood of true effect magnitude being substantial
			% difference	±90% CL	ES (*d*)	±90% CL		
	HIT+RT vs. MICT+RT	PRE-POST	-25	48	-0.43	0.90	small	possibly
		POST-+3 h	-29	35	-0.52	0.72	small	possibly
		PRE-+3 h	-47	34	-0.95	0.91	moderate	likely
**18S rRNA (span)**	HIT+RT vs. RT	PRE-POST	95	156	0.52	0.57	small	possibly
		POST-+3 h	15	102	0.11	0.62	trivial	unlikely
		PRE-+3 h	125	253	0.63	0.75	moderate	possibly
	MICT+RT vs. RT	PRE-POST	59	72	0.32	0.30	small	possibly
		POST-+3 h	26	88	0.16	0.45	trivial	unlikely
		PRE-+3 h	101	137	0.48	0.44	small	possibly
	HIT+RT vs. MICT+RT	PRE-POST	-18	61	-0.13	0.45	trivial	unlikely
		POST-+3 h	9	77	0.06	0.42	trivial	unlikely
		PRE-+3 h	-11	103	-0.07	0.64	trivial	unlikely
**28S rRNA**	HIT+RT vs. RT	PRE-POST	73	55	1.23	0.71	large	very likely
		POST-+3 h	14	60	0.28	1.13	small	possibly
		PRE-+3 h	97	115	1.52	1.24	large	likely
	MICT+RT vs. RT	PRE-POST	63	55	1.10	0.74	moderate	likely
		POST-+3 h	1	42	0.02	0.92	trivial	unlikely
		PRE-+3 h	65	76	1.12	1.00	moderate	likely

rRNA target	Comparison	Change between	Mean difference in change		Standardised effect size (ES)		Effect magnitude	Qualitative likelihood of true effect magnitude being substantial
			% difference	±90% CL	ES (*d*)	±90% CL		
	HIT+RT vs. MICT+RT	PRE-POST	-6	21	-0.18	0.69	trivial	possibly
		POST-+3 h	-11	47	-0.36	1.58	small	possibly
		PRE-+3 h	-16	52	-0.54	1.81	small	possibly
**28S rRNA (span)**	HIT+RT vs. RT	PRE-POST	123	109	0.81	0.48	moderate	likely
		POST-+3 h	24	70	0.22	0.54	small	possibly
		PRE-+3 h	153	136	0.93	0.52	moderate	very likely
	MICT+RT vs. RT	PRE-POST	58	66	0.50	0.45	small	possibly
		POST-+3 h	45	68	0.41	0.50	small	possibly
		PRE-+3 h	128	127	0.91	0.59	moderate	likely
	HIT+RT vs. MICT+RT	PRE-POST	-29	36	-0.65	0.92	moderate	possibly
		POST-+3 h	17	60	0.29	0.93	small	possibly
		PRE-+3 h	-10	58	-0.20	1.15	small	possibly

Measure	Comparison	Change between	Mean difference in change		Standardised effect size (ES)		Effect magnitude	Qualitative likelihood of true effect magnitude being substantial
			% difference	±90% CL	ES (*d*)	±90% CL		
**Type I fibre CSA**	HIT+RT vs. RT	PRE-POST	-34	22	-1.03	0.80	moderate	likely
	MICT+RT vs. RT	PRE-POST	-15	54	-0.39	1.45	small	possibly
	HIT+RT vs. MICT+RT	PRE-POST	29	86	0.63	1.53	moderate	possibly
**Type II fibre CSA**	HIT+RT vs. RT	PRE-POST	-15	23	-0.43	0.69	small	possibly
	MICT+RT vs. RT	PRE-POST	-2	32	-0.07	0.92	trivial	possibly

	HIT+RT vs. MICT+RT	PRE-POST	16	39	0.42	0.97	small	possibly
Maximal strength								
**1-RM leg press**	HIT+RT vs. RT	PRE-POST	-7.4	8.7	-0.40	0.40	small	likely
	MICT+RT vs. RT	PRE-POST	-8.2	9.9	-0.60	0.45	moderate	likely

SUPPLEMENTARY FILE B: Full-length Western blot images.

Manuscript title: Enhanced skeletal muscle ribosome biogenesis, yet attenuated mTORC1 and ribosome biogenesis-related signalling, following short-term concurrent versus single-mode resistance training.

Author list: Jackson J. Fyfe[1,2,3*], David J. Bishop[1,4], Jonathan D. Bartlett[1], Erik D. Hanson[1,5], Mitchell J. Anderson[1], Andrew P. Garnham[1,2] & Nigel K. Stepto[1,6,7].

Author affiliations:

1) Institute of Sport, Exercise and Active Living (ISEAL), Victoria University, Melbourne, Australia; 2) School of Exercise and Nutrition Sciences, Deakin University, Melbourne, Australia; 3) Centre for Sport Research (CSR), Deakin University, Melbourne, Australia; 4) School of Medical and Health Sciences, Edith Cowan University, Joondalup, Australia; 5) Department of Exercise and Sport Science, University of North Carolina at Chapel Hill, North Carolina, USA; 6) Monash Centre for Health Research and Implementation, School of Public Health and Preventive Medicine, Monash University, Melbourne, Australia; 7) Australian Institute for Musculoskeletal Science (AIMSS), University of Melbourne, Victoria University and Western Health, Sunshine Hospital, St Albans, Australia

Figure A. Full-length[1] Western blot images relating to data presented in manuscript Figure 4.

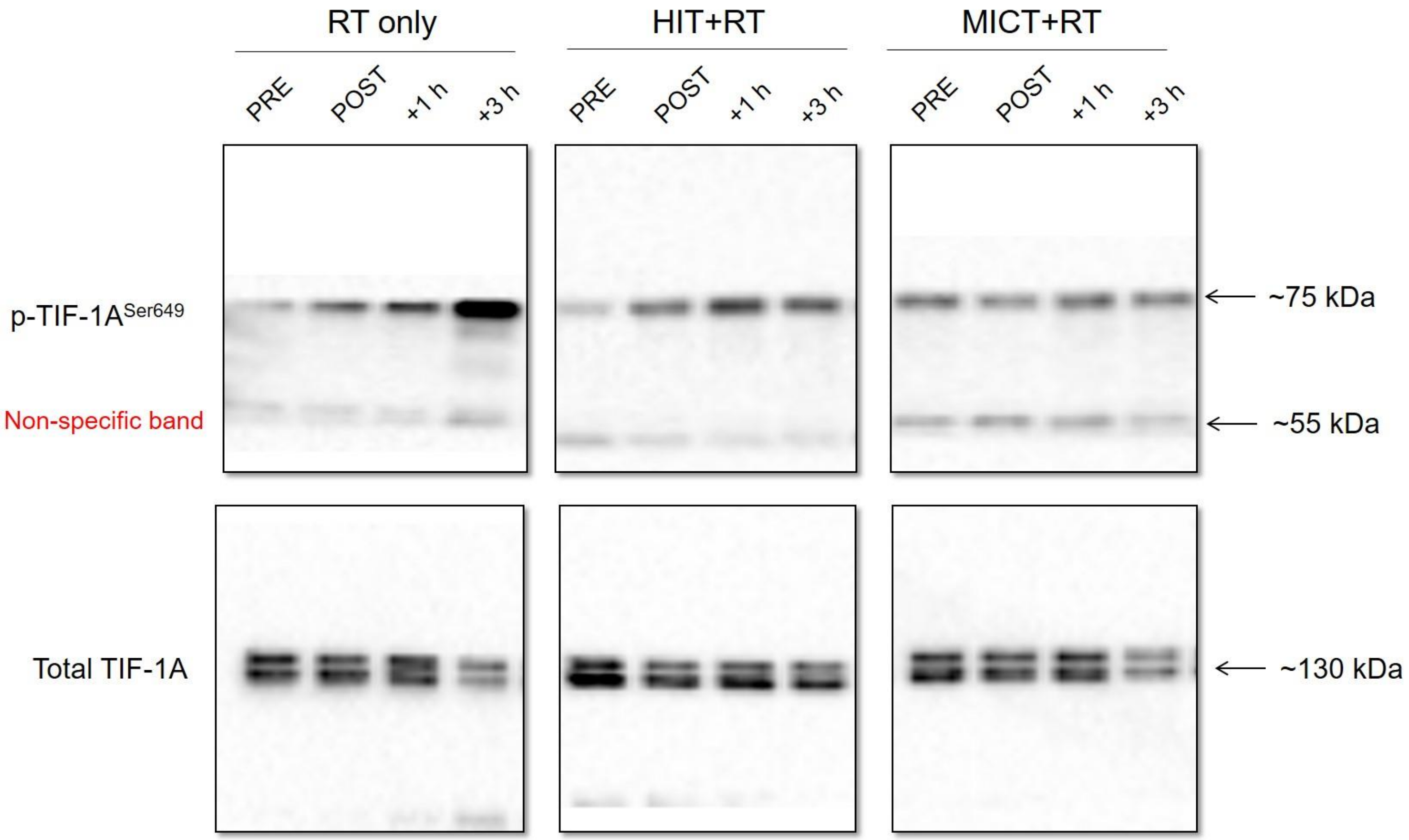

---

[1] After transfer, membranes were cut to approximately the vertical size displayed in the images above, to allow for probing with multiple primary antibodies on a single membrane/gel. The above images therefore display the largest available vertical membrane area probed with each respective primary antibody. Images for each protein and training group were obtained from separate gels, and are therefore separated for each training group to indicate this.

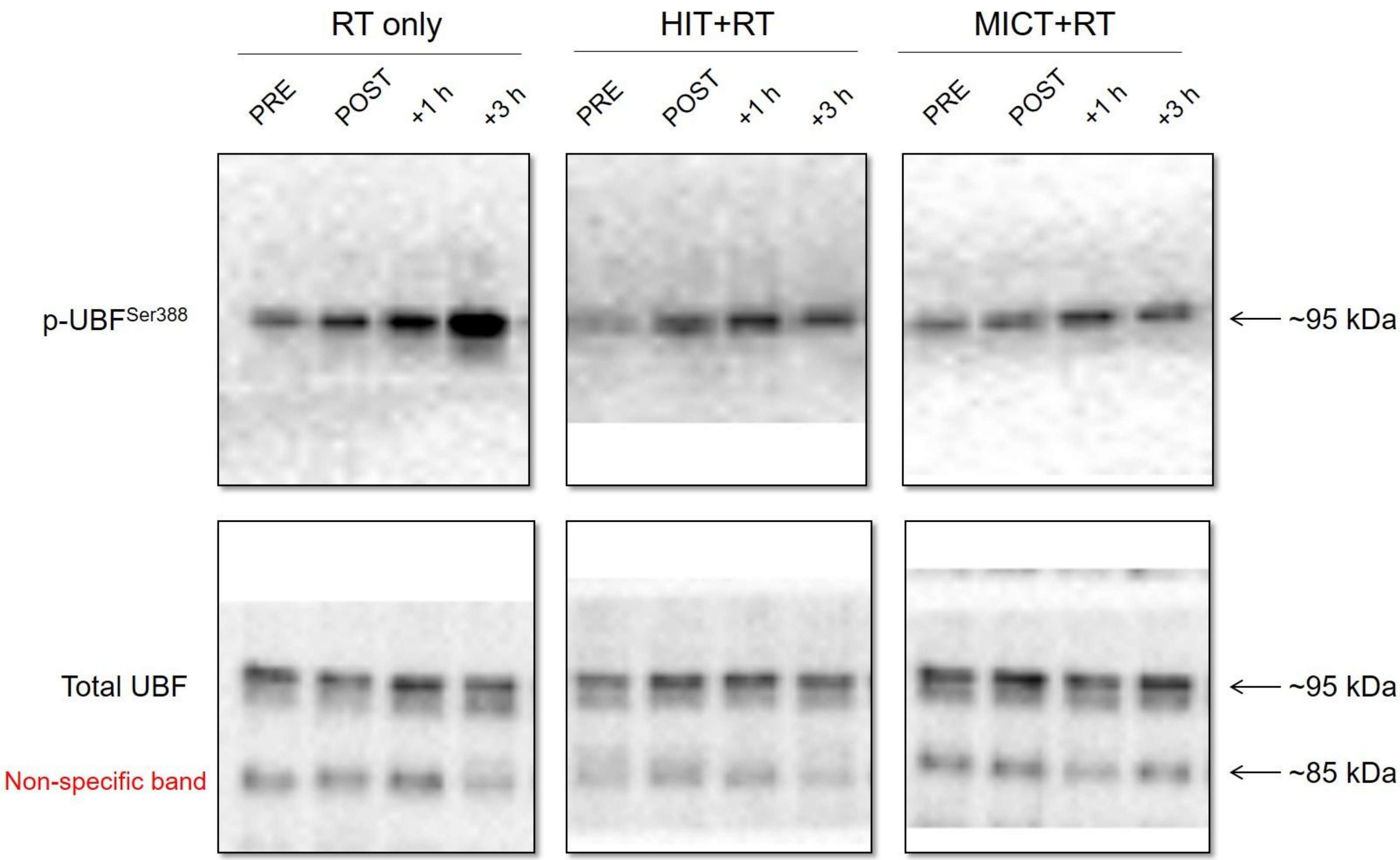
RT only
HIT+RT
MICT+RT
PRE
POST
+1 h
+3 h
p-UBF^Ser388
~95 kDa
Total UBF
Non-specific band
~95 kDa
~85 kDa

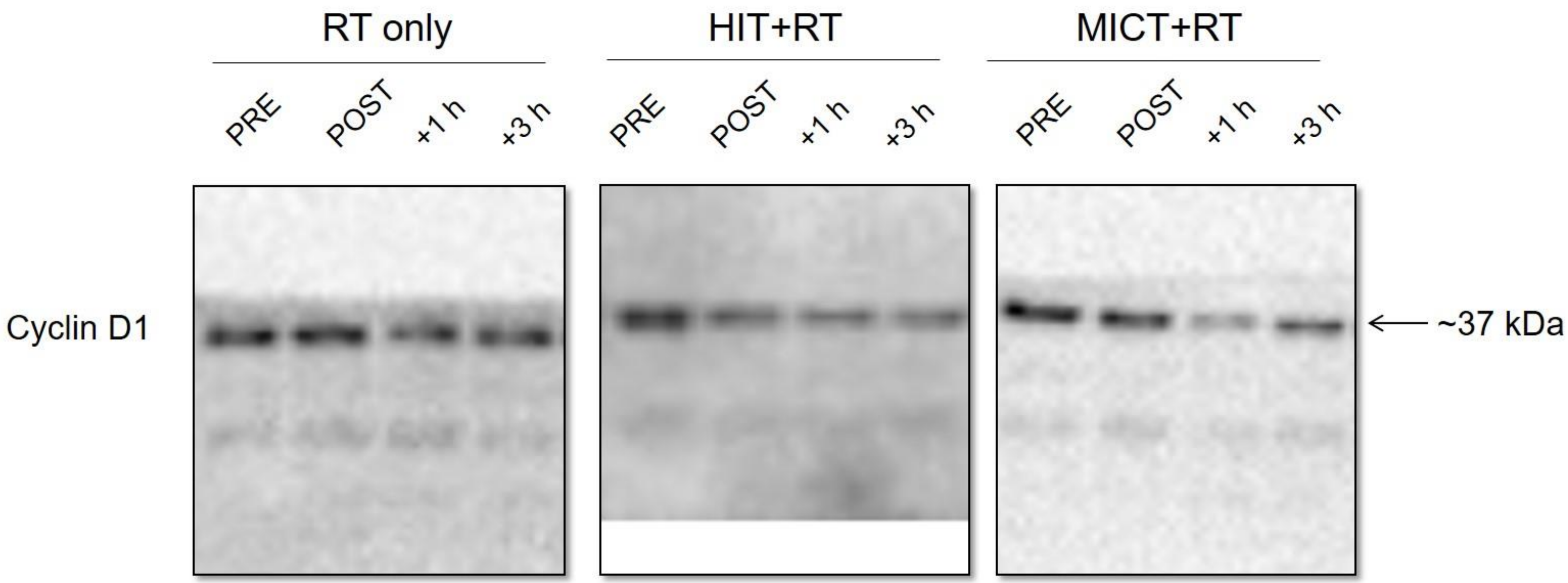
RT only
HIT+RT
MICT+RT
PRE
POST
+1 h
+3 h
PRE
POST
+1 h
+3 h
PRE
POST
+1 h
+3 h
Cyclin D1
~37 kDa

Figure B. Full-length[2] Western blot images relating to data presented in manuscript Figure 6.

[2] After transfer, membranes were cut to approximately the vertical size displayed in the images above, to allow for probing with multiple primary antibodies on a single membrane/gel. The above images therefore display the largest available vertical membrane area probed with each respective primary antibody. Images for each protein and training group were obtained from separate gels, and are therefore separated for each training group to indicate this.

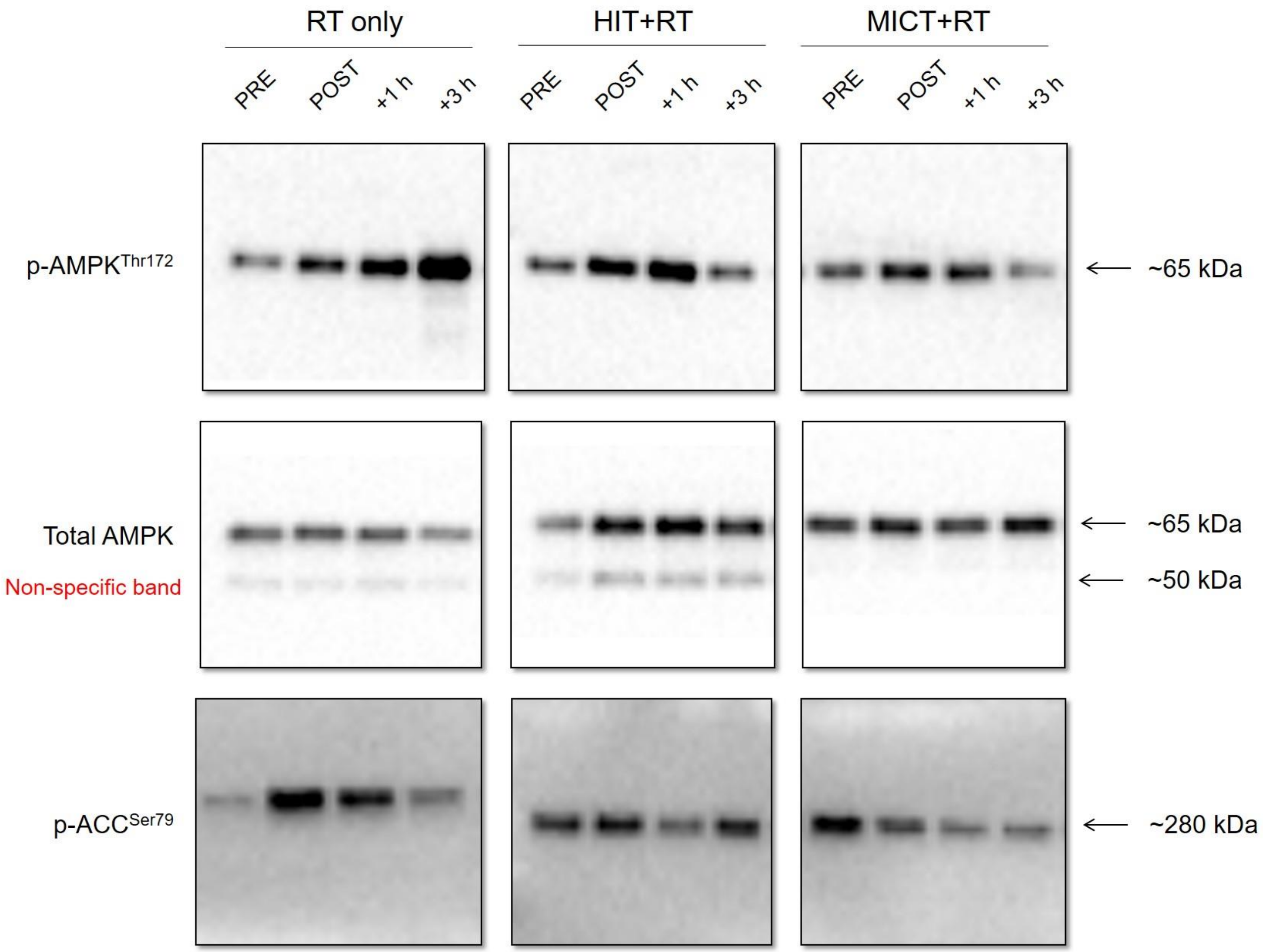
RT only
HIT+RT
MICT+RT
PRE
POST
+1 h
+3 h
p-AMPK^Thr172
Total AMPK
Non-specific band
p-ACC^Ser79
~65 kDa
~65 kDa
~50 kDa
~280 kDa

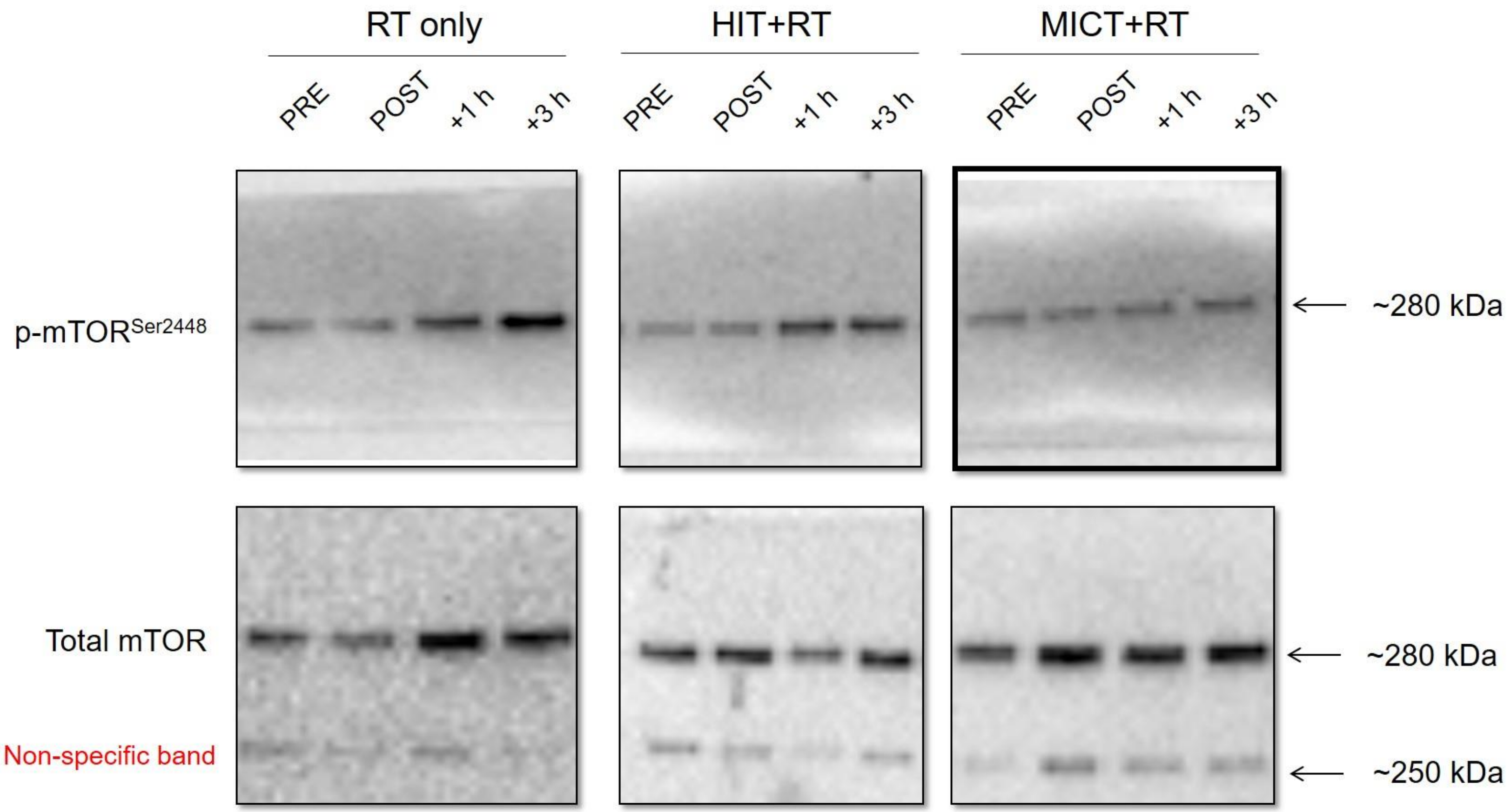
RT only
HIT+RT
MICT+RT
PRE
POST
+1 h
+3 h
PRE
POST
+1 h
+3 h
PRE
POST
+1 h
+3 h
p-mTOR^Ser2448
~280 kDa
Total mTOR
~280 kDa
Non-specific band
~250 kDa

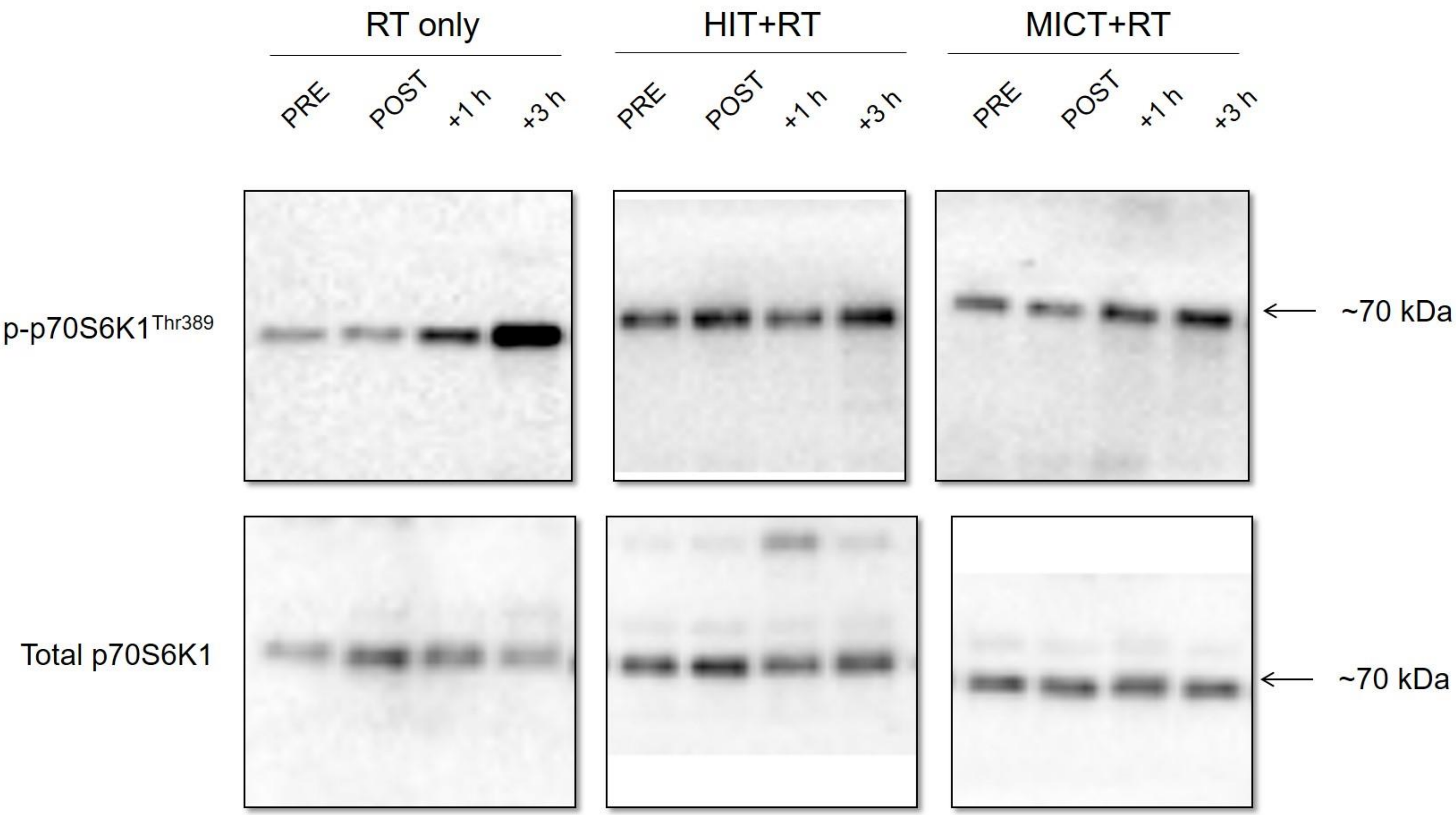
RT only
HIT+RT
MICT+RT
PRE
POST
+1 h
+3 h
p-p70S6K1Thr389
Total p70S6K1
~70 kDa
~70 kDa

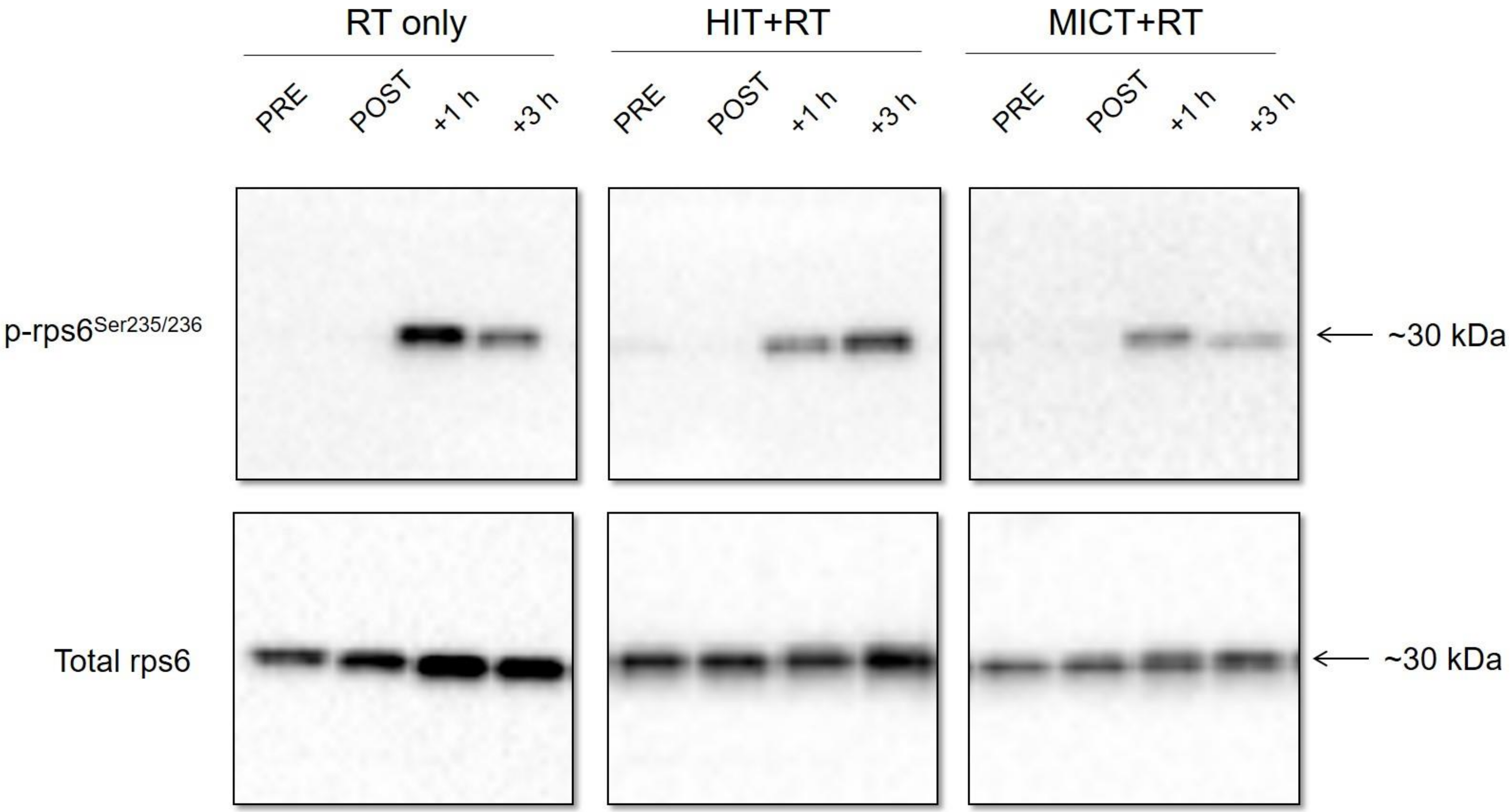
RT only
HIT+RT
MICT+RT
PRE
POST
+1 h
+3 h
PRE
POST
+1 h
+3 h
PRE
POST
+1 h
+3 h
p-rps6Ser235/236
~30 kDa
Total rps6
~30 kDa

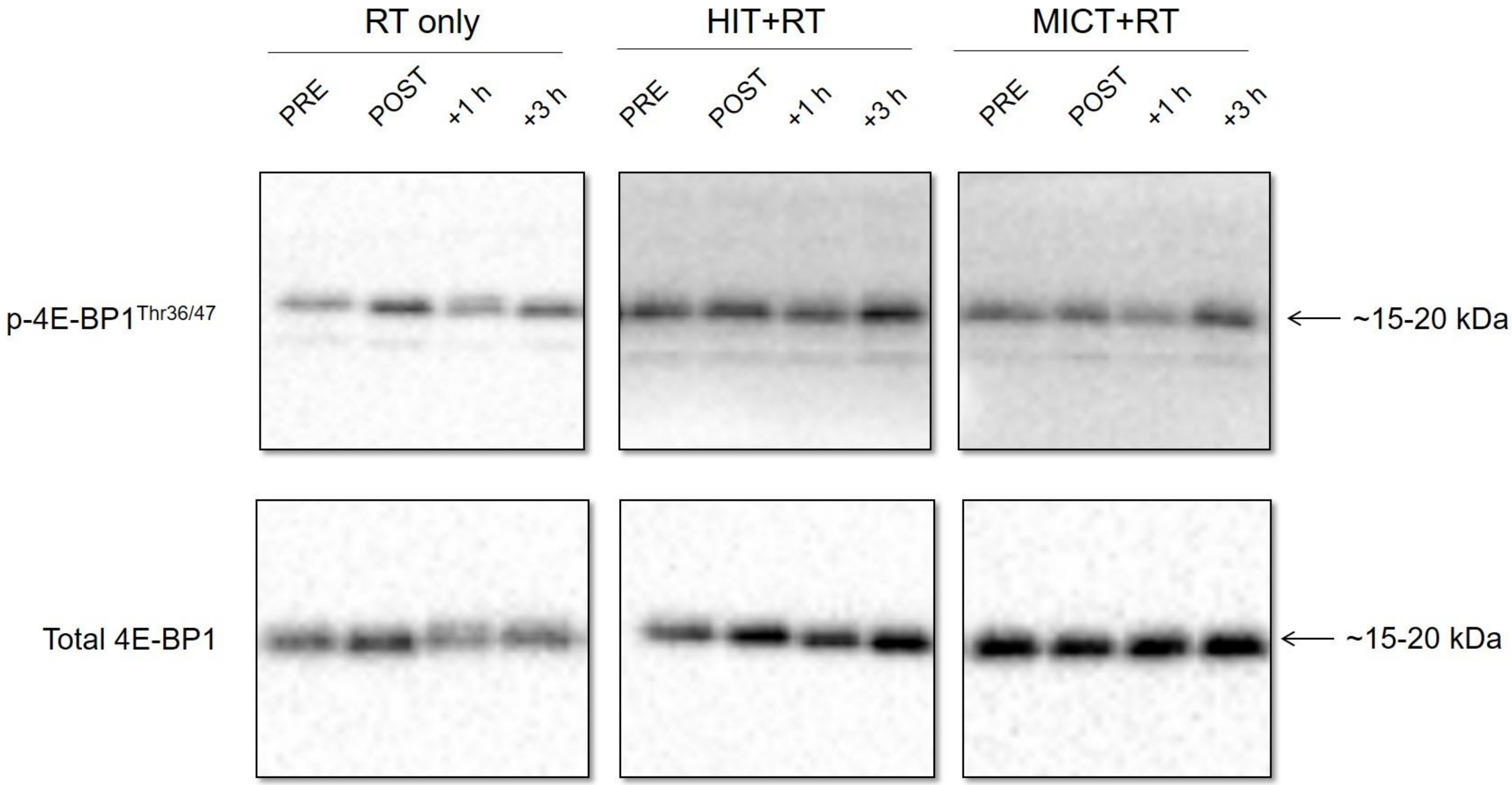
RT only
HIT+RT
MICT+RT
PRE
POST
+1 h
+3 h
p-4E-BP1Thr36/47
Total 4E-BP1
~15-20 kDa
~15-20 kDa
